# Editorial Notes

**Published:** 1910-12

**Authors:** 


### Editorial Notes.

**The University of Bristol.**

THE following communication has been received from a well-known graduate of one of the older Universities. We venture to think that it demands the serious consideration of the University authorities, and publish it in the hope that it may have a good influence in preventing the recurrence of such unseemly demonstrations as marred the last conferment of degrees.

"The first brood of graduates of Bristol University has now flown from the nest. The degrees were conferred in the large hall of the Victoria Rooms on the 20th of October, in the presence of a crowded audience, with a ceremonial which was devised, and deserved, to impress by its magnificence and solemnity. That it failed to do so can be gathered from the testimony of all who were present. And the reason should be frankly analysed for the sake of the fair name and well-being of the University, as well as for the comfort of those who may be attracted to similar ceremonies in future years. A good number on this occasion left with a bad headache.

"The rooms were packed to their fullest capacity with all that was representative of Bristol in its civic, social, commercial, and learned components. The galleries were as crowded as the floor. Distinguished visitors from all parts of England had come to show their benevolent interest in the youngest of English Universities. Beneath the organ and around it, on the rising benches, in the focus of all eyes, were massed the students, attracting attention not only by the noise they made, but by the devices, emblems, and inscribed banners they displayed and waved. Some were travestied in masks and carnival costumes. From the first they kept up a continual noise, which drowned the powerful organ, reduced the speeches to an inaudible pantomime, and prevented a spectator from communicating with his neighbour except by shouting in his ear.

Even when the official proceedings commenced this noise went on without stint or stay; from that moment there were two rival organisations in the field. The authorities triumphed in the sense that they carried their programme through. They worked, though in dumb show, with unflurried steadiness, and as a result the degrees were well and truly conferred.

"Benignant toleration was the attitude of the powers, and for a time that of the audience at large. Next day the local newspapers took a line of genial banter, and embroidered, in not unfriendly spirit, on the text that 'boys will be boys.'

"But it is a question whether the University itself—by which term we include students—would act wisely in letting things rest there. No doubt there is a good deal to be said for doing nothing: 'Young blood will have its course,' and 'Youth will be served.' The policy of 'quieta non movere' is a seductive one, and not likely to languish for want of supporters, so we make no apology for offering a few considerations on the other side.

"That little unfavourable comment has been heard, or rather seen in print, is no evidence that the misbehaviour was generally well liked or even condoned. The local newspapers, animated by feelings of local patriotism, kept their finger on the throttle valve. It is only under a serious sense of responsibility that we venture to speak somewhat bluntly about what we know. And we have no doubt, after inquiry, that large and important sections of the audience in the Victoria Rooms felt indignation and disgust. The Town Councillors were among these. It is not likely that civic dignitaries would care to assemble in the pageantry of their official robes, and in the presence of their wives and constituents, merely to play second fiddle in a pandemonium. The University, it should be remembered, depends on these dignitaries for a good part of its grants; if their respect and approval come to be forfeited the grants are likely to be cut down as well. We feel bound in this connection to emphasise the fact that the University is, to put it baldly, a bounty-fed institution. The students should hardly need to be reminded that they gain very considerable educational advantages at a fraction of the prime cost, thanks to the generosity of certain broad-minded and wealthy citizens. Such of these as were present at the degree giving would be poorly impressed with the students' corporate gratitude and sense of fitness in nullifying completely a ceremony that was tended to be impressive and symbolic of a great and generous aspiration. When everyone else in the hall had come with the amiable desire to give the University a good send off—ladies in their prettiest dresses, officials in their robes, professors primed with speeches prepared with felicitous care for delivery in English—there was a jarring note of selfishness in the behaviour of those who stultified all these preparations by humouring their own instincts of organised disorder. The programme was, indeed, 'butchered to make a Roman holiday.'

"At the best and oldest Universities such practices, it is needless to say, would not be tolerated, and we must add, in fairness, they are not even attempted. 'Rags' there are at both Cambridge and Oxford, but the participants have at least the saving grace to recognise what times and seasons are suitable and what are not. Bristol has special reasons for forming a good tradition, and for beginning it early. We admit that no hard and fast line can be drawn where the instinct of propriety is concerned, as human nature must be considered, and human nature is a good thing in the main. But when it comes to riotous disorder on the part of a couple of hundred persons, in the presence of a couple of thousand unwilling and powerless spectators, we are past all sane limits. Someone must draw the line, and the question is who? If the students can draw it for themselves no more excellent way could be found. But if they fail we venture to suggest that the authorities should co-operate with them. It is unnecessary to give our own idea of the best way to set matters right, for that surely may be left to the good sense of those in authority. But no travesties, masks, confetti, missiles, banners, drums, trumpets, or other concrete paraphernalia of disorder should be allowed. Noise should cease during the actual proceedings. Of course, there is no objection to spontaneous wit or special recognition of popular favourites—in fact, these give light and shade to the ceremony, like the play of summer lightning over the hills of a severe landscape. But all this should be auxiliary to the ceremony, and arise out of it. The complaint we have to make, in common with the public, is that there was no ceremony; it was smothered, drowned, and extinguished by a monotonous, senseless, and incessant din."

**State Sickness Insurance.**

For a long time, we might say always, medical attendance on the working-classes and the poor has been unsatisfactory—unsatisfactory to the profession both from the medical and from the business point of view, whether in private or club practice, under the Poor Law, or in hospital; unsatisfactory to the public, though they have not as fully realised the position, and have been more or less content with inefficient medicine just as they were with bad sanitation, indifferent food and deficient education. But now they are waking up to the need of something better, and reports of various inquiries into the health and care of the people, especially the report of the Poor Law Commission, have revealed to the public conditions in the present system of medical attendance which are "inherently inimical to the cure and prevention of disease," and the State has determined to intervene. The Government has drawn up a scheme of insurance against sickness and invalidity, in which we take it for granted that some provision is made for the medical attendance of those insured. As the Government is going to undertake part, at any rate, of the risk of the insurance, it will be to its interest to provide the best attendance, in order that those insured may be treated in the most efficient manner, and so cured as quickly as possible. It stands to reason that any such scheme must affect most medical men, whether general practitioners, Poor Law officers, specialists, or members of a hospital staff, but especially those who practice among the working-classes and those who have undertaken contract practice. That the profession in Bristol and the neighbourhood fully appreciate the importance of the question must have been evident to any-one who was present at the large meetings held to consider it in October.

There can be no doubt that medical men are utterly opposed to a continuance of the old system of contract practice, with all its abuses, lay control, insufficient remuneration, absence of wage limit, its practice of giving no free choice of doctor, and the feeling that under it medical men were ever at the beck and call of their patients without any means of protection from abuse of their services under the contract. The profession has now to make up its mind as to how it wishes the evils of this sort of practice to be remedied. Shall the State devise the remedy with the advice and the co-operation of the profession, or shall the profession devise the remedy, or the State alone do it, and the profession merely offer for purchase its services as medical attendant? In any case it is essential and in accordance with the past policy of the British Medical Association that the profession should act collectively and not individually, whether in advising or co-operating with the Government, in carrying out its own schemes or in bargaining with the State or the sale of its services; and in the event of the Government plans for any reason failing, we must be prepared to carry out ourselves some scheme for the betterment of the conditions of practice among the working-classes. Whatever course is adopted, it appears to be generally agreed that the patient shall have free choice of doctor, and that the remuneration shall be sufficient for the proper conduct of a medical man's practice. It is interesting here to note the remark made at one of the meetings referred to above: it was, "Medical fees are based not on the intrinsic value of the services rendered, but on the average amount which it is necessary to charge in order to produce an adequate income from the number of visits which a medical practitioner can conscientiously make." From this it appears that what is aimed at is not the fixing of a definite fee or scale of fees for each attendance, but of a sum of money which shall be properly proportioned to the amount of work done. Nevertheless, a great difference of opinion exists as to the method of payment: whether the doctor shall be paid a certain fixed sum per annum for each person that he may be liable to attend, or whether he shall be paid a fee for each separate attendance as in private practice. The first system has been advocated largely because of its simplicity in working, and partly because, until lately at any rate, no practicable scheme had been produced for the working of the other system. It is argued that with a proper fee, with professional control, with a larger wage limit, and with free choice of doctor, the conditions of such a contract would be totally different to the conditions of old club practice, and would be advantageous to the profession. If a medical man were paid so much per head, he would be much in the position of a man with a regular salary, with this difference, that his salary would vary directly in proportion to the amount of work he might reasonably be considered liable to have to do, and he will have a fairly good idea of what his income will be each year. There is said to be great difficulty in estimating what a fair capitation fee should be, but some idea of it might be got by estimating the value of private practice in unopposed districts, where the population liable to attendance would be known, excluding of course Poor Law and contract practice patients. Say that with such a proportion of a thousand a man estimated his private practice at £500 per annum, then the fair fee would be 10s. per head. However, it appears that even such remuneration as this would not be acceptable to the supporters of the system of "payment for work done." Their contention is that the medical man should not take the risk of the insurance which he does under the other system, that the State is insuring the patient against sickness and should provide the necessary medical attendance, just as any insurance company provides for any loss that it may have insured against, that such a method of payment will be much more equitable and satisfactory, and that a medical man will know that he is being paid exactly in proportion to the amount of work that he does; that the method of paying for each attendance has been adopted by the British Medical Association in its scheme for school clinics, and that it has been adopted abroad with success. Also that under such a system the man who succeeds in practice can be rewarded as he should be, not by more patients, but by better fees, and so more time in which to do his work, for he can always arrange with his patients to accept the payments made for them by the Government as part of his fees. In this scheme no wage limit would be necessary if the fee was, as it should be, an adequate one; but it appears to us that under any Government scheme the question of a wage limit would naturally be settled by the Government, who would take care to protect themselves from abuse.

We cannot get away from the practical difficulty. Will any Government undertake such an uncertain risk as the payment of the doctors' bills for fifteen millions of people? It is a risk that could only be undertaken by the State or some body with a large reserve fund. We cannot recall any department in which they do anything of the sort; and their salaried medical officers accept, and do not seem to object to much larger risks than the man paid fees per capita—the latter's pay does vary in direct proportion to the number he is liable to attend, the former's salary does not.

It is admitted that some means would have to be taken to prevent abuse by medical men, who consciously or unconsciously, if paid for each attendance, would be liable to give more attendance than they otherwise would. We do not think that any Government would give a free hand to every medical man and to every fanciful or fussy patient. The question of what these safeguards against over-attendance should be is a most difficult one, and is beset with two serious difficulties—one, that the safeguards may by their very nature interfere with the very essence of the Government scheme, which is the prevention of sickness by the provision of prompt, free, and unrestricted medical attendance; the other, that the methods of inspection and inquiry adopted might be quite intolerable to the medical profession.

If a medical man is careful in diagnosis and prompt and skilful in treatment, he will pay many less visits, and if he is paid for each visit he will actually penalise himself, and it does not follow of necessity that he will be compensated by popularity and more patients.

It is very evident that there is great need for discussion and criticism by the profession, and we expect the criticism of a learned profession to be scientific and not polemical. It will be necessary in these discussions to remember that we have to find not only what is desirable, but what is practicable; that if we are blinded by our ideals, and if our demands are unreasonable, we may not only be left where we were, and from where we have long been trying to get away, but may be chained there by some objectionable Government scheme being thrust on us.

We must give whatever schemes are presented to us our most careful consideration, and do our utmost to perfect whichever we select, and it is essential that it should have the unanimous support of the profession; but to succeed it must not only be agreeable to the profession, but acceptable to the Government, and that will be most acceptable to the Government which is most convenient, and that will be most convenient which is backed up by the irresistible force of an enthusiastic and unanimous profession. Although we think that the system of payment per head is suffering unjustly from prejudice and from the sins of its ancestors, we have a feeling that the principle of payment for work done is the one most likely to obtain unanimity and enthusiasm, because it appears so much more attractive.

